# **‘**Fertile island’ effects on the soil microbial community beneath the canopy of *Tetraena mongolica*, an endangered and dominant shrub in the West Ordos Desert, North China

**DOI:** 10.1186/s12870-024-04873-4

**Published:** 2024-03-08

**Authors:** Zhangkai Liu, Yuying Shao, Qingguo Cui, Xuehua Ye, Zhenying Huang

**Affiliations:** 1 State Key Laboratory of Vegetation and Environmental Change, Institute of Botany, Chinese Academy of Science, Beijng, 100093 China; 2 University of Chinese Academy of Science, Beijng, 100049 China; 3 China National Botanical Garden, Beijing, 100093 China

**Keywords:** Desert ecosystem, Fertile island effect, Functional diversity, Soil microbial, *Tetraena mongolica*

## Abstract

**Background:**

The fertile islands formed by shrubs are major drivers of the structure and function of desert ecosystems, affecting seedling establishment, plant–plant interactions, the diversity and productivity of plant communities, and microbial activity/diversity. Although an increasing number of studies have shown the critical importance of soil microbes in fertile island formation, how soil microbial community structure and function are affected by the different fertile island effect intensities is still unknown. As an endangered and dominant shrub species in the West Ordos Desert, *Tetraena mongolica* was selected for further exploration of its fertile island effect on the soil microbial community in the present study to test the following two hypotheses: (1) *T. mongolica* shrubs with different canopy sizes exert fertile island effects of different strengths; (2) the soil microbial community structure and function beneath the *T. mongolica* canopy are affected by the fertile island, and the strength of these effects varies depending on the shrub canopy size.

**Results:**

The contents of soil total nitrogen (TN) and available phosphorus (AVP) were significantly greater beneath *T. mongolica* shrub canopy than outside the shrub canopy. With increasing shrub canopy size, the enrichment of soil TN and AVP increased, indicating a stronger fertile island effect. The structure and function of soil microbial communities, including fungal, archaeal and bacterial communities, are affected by the fertile island effect. An increase in canopy size increased the relative abundance of Ascomycota (Fungi) and Thaumarchaeota (Archaea). For the soil microbial functional groups, the relative abundance of endophytes in the fungal functional groups; steroid hormone biosynthesis, sphingolipid metabolism, and steroid biosynthesis genes in the bacterial functional groups; and nonhomologous end-joining and bisphenol degradation functional genes in the archaeal functional groups increased significantly with increasing *T. mongolica* canopy size.

**Conclusions:**

These results revealed that *T. mongolica* had a fertile island effect, which affected the soil microbial community structure and functions, and that the fertile island effect might increase with increasing shrub canopy size. The fertile island effect may strengthen the interaction between *T. mongolica* shrubs and microbes, which may be beneficial to the growth and maintenance of *T. mongolica*.

**Supplementary Information:**

The online version contains supplementary material available at 10.1186/s12870-024-04873-4.

## Introduction

Deserts cover approximately 30% of the world's land area [1]. The total area of desert ecosystems in China is approximately 1.65 × 10^6^ km^2^, accounting for 17% of the total land area [2]. In desert ecosystems, for example, the western Ordos desert in China, the dominant species in plant communities are mostly woody plants. The western Ordos desert is a centre of biological diversity for rare and endangered shrubs in China's desert regions, hosting approximately 300 species of shrub plants [3–5]. However, due to changes in land use type, industrial pollution and other human activities, as well as the stress of harsh environments, desert ecosystems are facing severe and persistent threats, and the distribution areas of rare plants are gradually shrinking and becoming disconnected [6, 7]. There are many rare and endangered shrub species distributed in the western Ordos desert, including *Tetraena mongolica*, *Ammopiptanthus mongolicus*, and *Helianthemum songaricum* [3, 5]. Desertification and climate change are among the major ecological and environmental issues faced by these rare and endangered shrub species and have resulted in biodiversity loss in this region [5]. It is projected that the desertification area under representative concentration pathways (RCPs) RCP8.5 and RCP4.5 will increase by 23% and 11%, respectively, by the end of the twenty-first century relative to the 1961–1990 baseline, which could pose an enormous threat to desert vegetation, especially for constructive species [8, 9].

Desert vegetation is sparsely distributed with low shrub coverage. The presence of shrubs enriches soil resources under their canopies, forming so-called ‘fertile islands’ [10, 11]. Fertile islands might affect the spatial distribution and cycling of nutrients [12, 13], thereby affecting seedling establishment, plant–plant interactions, the diversity and productivity of plant communities, and microbial activity/diversity [14–18]. As an endangered but dominant shrub species, *T. mongolica* can play an important role in maintaining and improving the fragile desert ecosystem in the western Ordos desert, as well as in ensuring the balance of regional ecology and the maintenance of species diversity [19–21]. Previous studies have shown that *T. mongolica* shrubs can effectively accumulate soil nutrients, improve soil texture and form typical fertile islands [22]. However, the fertile island effects of *T. mongolica* shrubs on soil microbes are not fully understood.

Soil microbes are important components of ecosystems and play crucial roles in regulating ecosystem functions and features, such as improving nutrient cycling, maintaining soil fertility, responding to climate change, and sequestering carbon [23–26]. Soil microbes can improve nutrient availability by degrading plant litter or residue and increasing nutrient uptake efficiency through reciprocity (e.g., mycorrhizal fungi) [27, 28]. The shrubs in desert ecosystems form fertile islands, which provide a ‘better’ (dark and wet) environment and rich carbon sources for decomposers [29, 30]. The abundances of fungi and bacteria beneath shrub canopies are reportedly greater than those in adjacent open areas lacking vascular vegetation in arid regions [31, 32]. Therefore, the properties of microbial communities can directly (for example, through nutrient fixation, litter decomposition, and organic matter mineralization) and indirectly (for example, through nutrient redistribution via fungal networks) affect the ability of plant patches to capture and recycle nutrients, thereby enhancing the fertile island effect [33, 34]. The strength of the fertile island effect may be closely related to the size of the shrub canopy [35]. Shrubs with larger canopies have greater ability to intercept litter and greater ability to improve soil and light conditions beneath the canopy [36]. This means that large scrub areas may have a stronger fertile island effect and thus may have a greater impact on microbes. An increasing number of studies have shown the critical importance of soil microbes in the formation of fertile islands [13, 37–43]; however, the role of soil microbes in the formation of fertile islands of different sizes has also not been fully studied.

In this study, we examined the fertile island effects of *T. mongolica* shrubs of different canopy sizes on the soil microbial community structure and function in a desert ecosystem in western Ordos, China, to test the following two hypotheses: (1) *T. mongolica* shrubs with different canopy sizes exert fertile island effects of different strengths; (2) the soil microbial community structure and function beneath the *T. mongolica* canopy are affected by the fertile island formed by *T. mongolica* shrubs, and the strength of these effects varies depending on the canopy size of the shrub. Studying the impacts of the fertile island effect exerted by *T. mongolica* shrubs on soil and soil microbes would be helpful for understanding the relationships between *T. mongolica* shrubs and soil microbes, as well as the growth strategies of *T. mongolica*, and provide a theoretical basis for the protection of rare plant biodiversity and desertification control and even for maintaining the stability of desert ecosystems.

## Results

Among the 30 different sizes of *T. mongolica*, the range of the shrub canopy was from 0.013 m^2^ to 4.081 m^2^, with an average of 0.619 m^2^. The contents of soil total potassium (TK) ranged from 16.99 to 20.89 g/kg, total phosphorus (TP) from 302.64 to 421.38 mg/kg, soil total nitrogen (TN) from 0.262 to 0.581 g/kg, soil total carbon (TC) from 6.61 to 22.77 g/kg, soil organic carbon (SOC) from 1.92 to 6.56 g/kg, soil inorganic carbon (SIC) from 3.67 to 17.56 g/kg, soil available phosphorus (AVP) from 2.54 to 16.85 mg/kg, and soil available potassium (AVK) from 88.92 to 238.05 mg/kg; soil pH ranged from 8.59 to 9.59. The specific data for each shrub is shown in Table S1.

### The fertile island effects of T. mongolica shrubs on soil nutrients

**Fig. 1:**
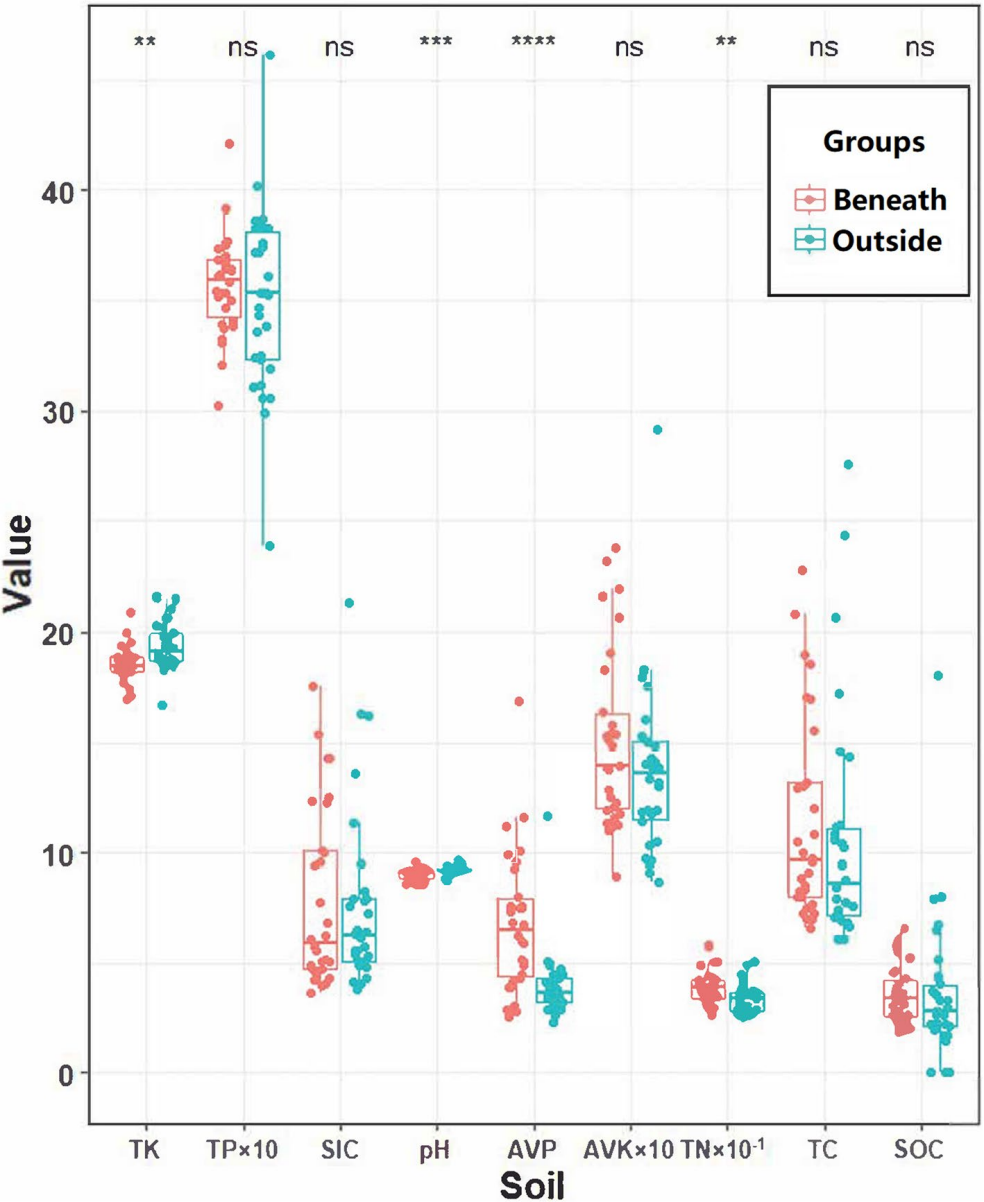
Soil properties beneath and outside the *T. mongolica* canopy. The units of TP, AVK, and AVP are mg/kg, and the units of TK, SIC, TN, TC, and SOC are g/kg. * indicates a significant difference at *p* < 0.05; ** indicates a significant difference at *p* < 0.01; *** indicates a significant difference at *p* < 0.001; ns indicates no significant difference

The soil TN and AVP beneath the shrub canopy were significantly greater than those outside the canopy, while the soil TK and pH beneath the canopy were significantly lower than those outside the canopy (Fig. 1, Table S2).

**Fig. 2:**
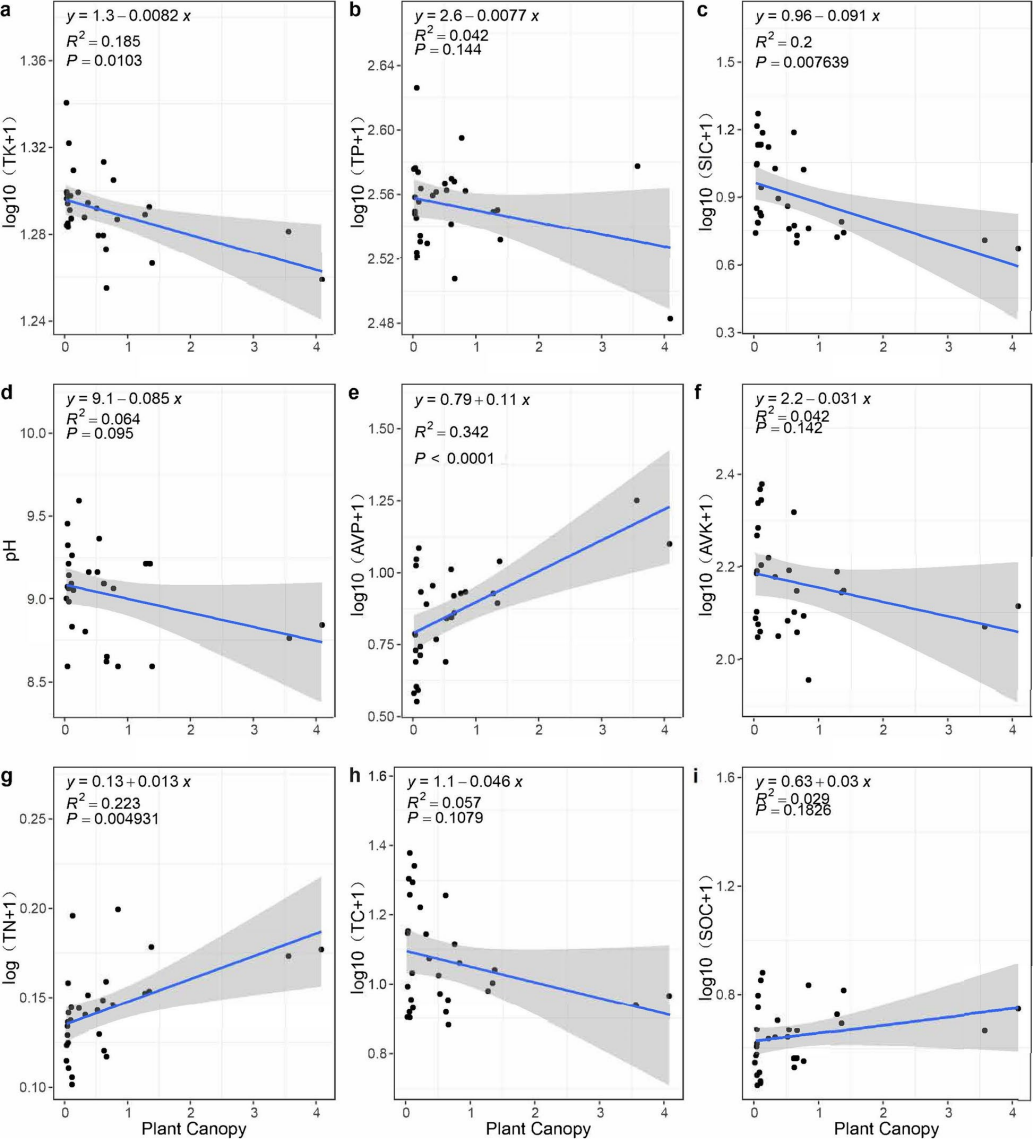
Linear regression analysis between *T. mongolica* canopy size and soil nutrients

With increasing canopy size, the soil TN and AVP significantly increased, while the soil TK and TIC significantly decreased. The soil TP, pH, AVK, TC, and SOC did not significantly change with increasing canopy size (Fig. 2).

### The fertile island effect of T. mongolica shrubs on the soil microbial community composition

**Fig. 3:**
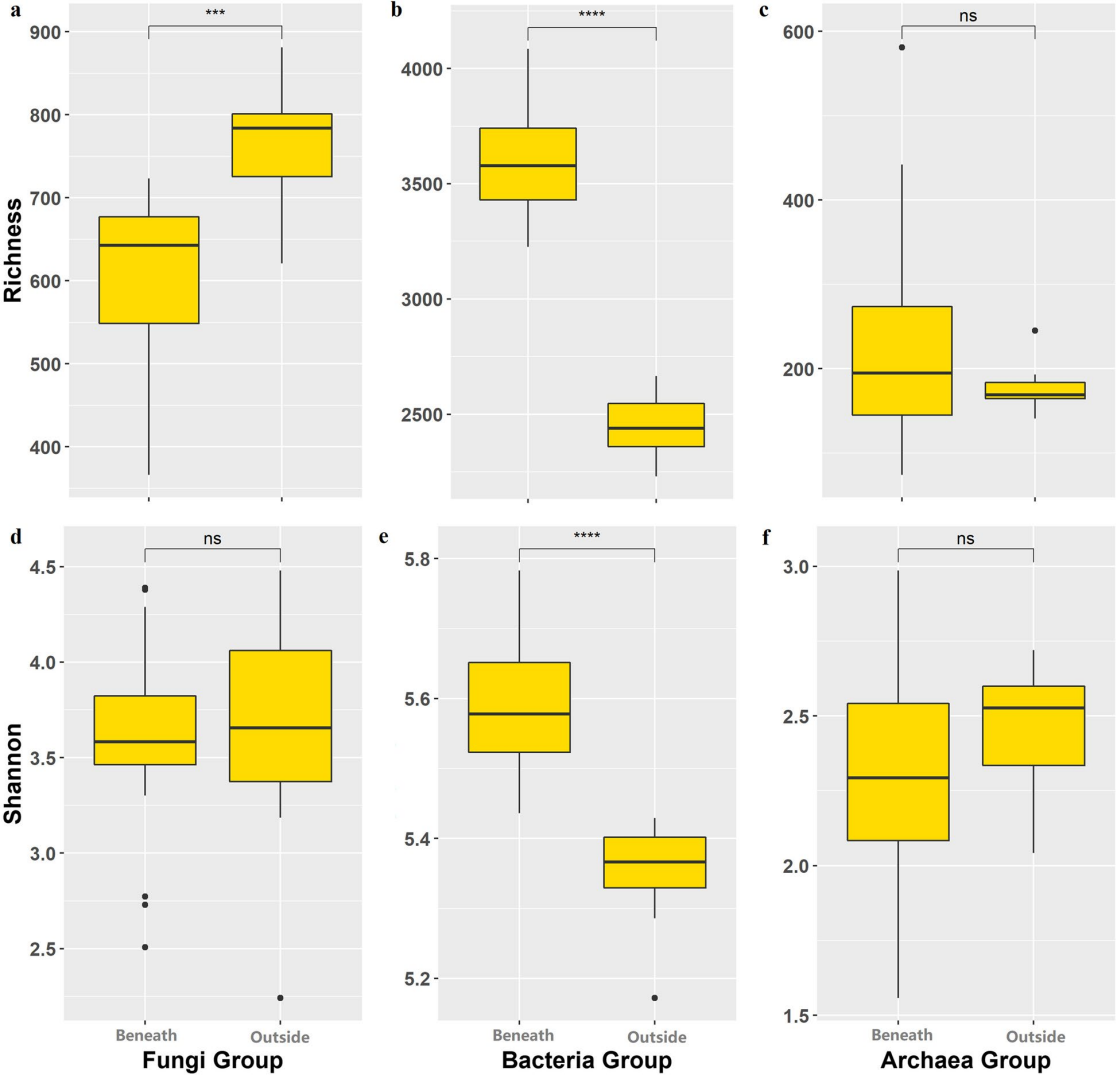
Richness and Shannon indices of the different soil microbial groups beneath and outside the *T. mongolica* canopy. *** indicates a significant difference at *p* < 0.001 between the inside and outside canopies; **** indicates a significant difference at *p* < 0.0001; ns indicates no significant difference

The fungal richness beneath the shrub canopy was significantly lower than that outside the shrub canopy, while there was no significant difference in the fungal Shannon index beneath and outside the shrub canopy; the bacterial richness and Shannon index beneath the shrub canopy were significantly greater than those outside the shrub canopy; and there was no significant difference in the archaeal richness or Shannon index beneath and outside of the shrub canopy (Fig. 3).

**Fig. 4:**
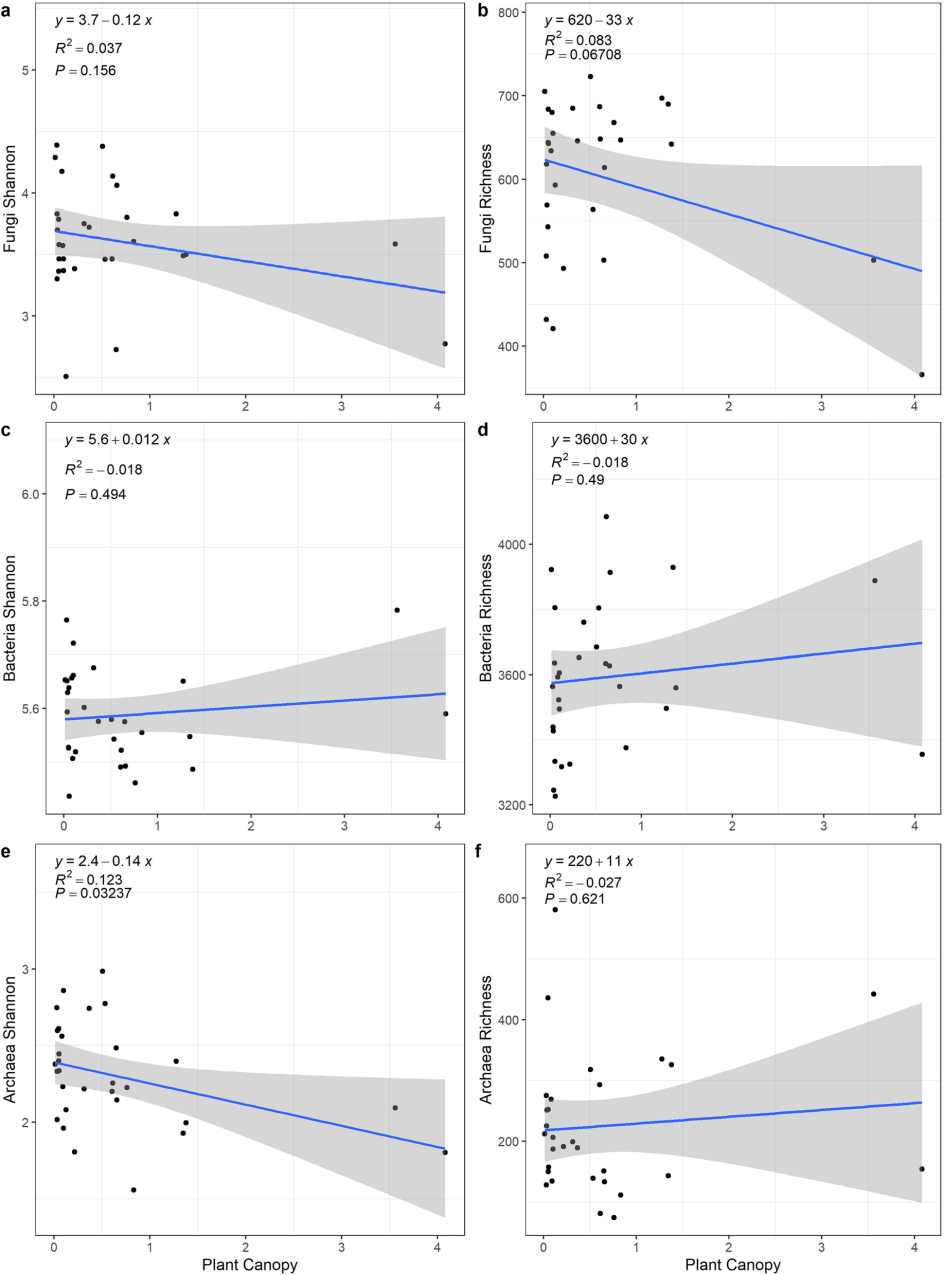
Linear regression between plant canopy size and the relative abundance of the major soil microbial communities. Panels **a** and **b** are the major phyla of the fungal community, panels **c** and **d** are the major phyla of the bacterial community, and panels **e** and **f** are the major phyla of the archaeal community

With increasing shrub canopy size, the richness of the fungi significantly decreased (*p* < 0.1), and the Shannon index of the archaea significantly decreased (*p* < 0.05) (Fig. 4).

**Fig. 5:**
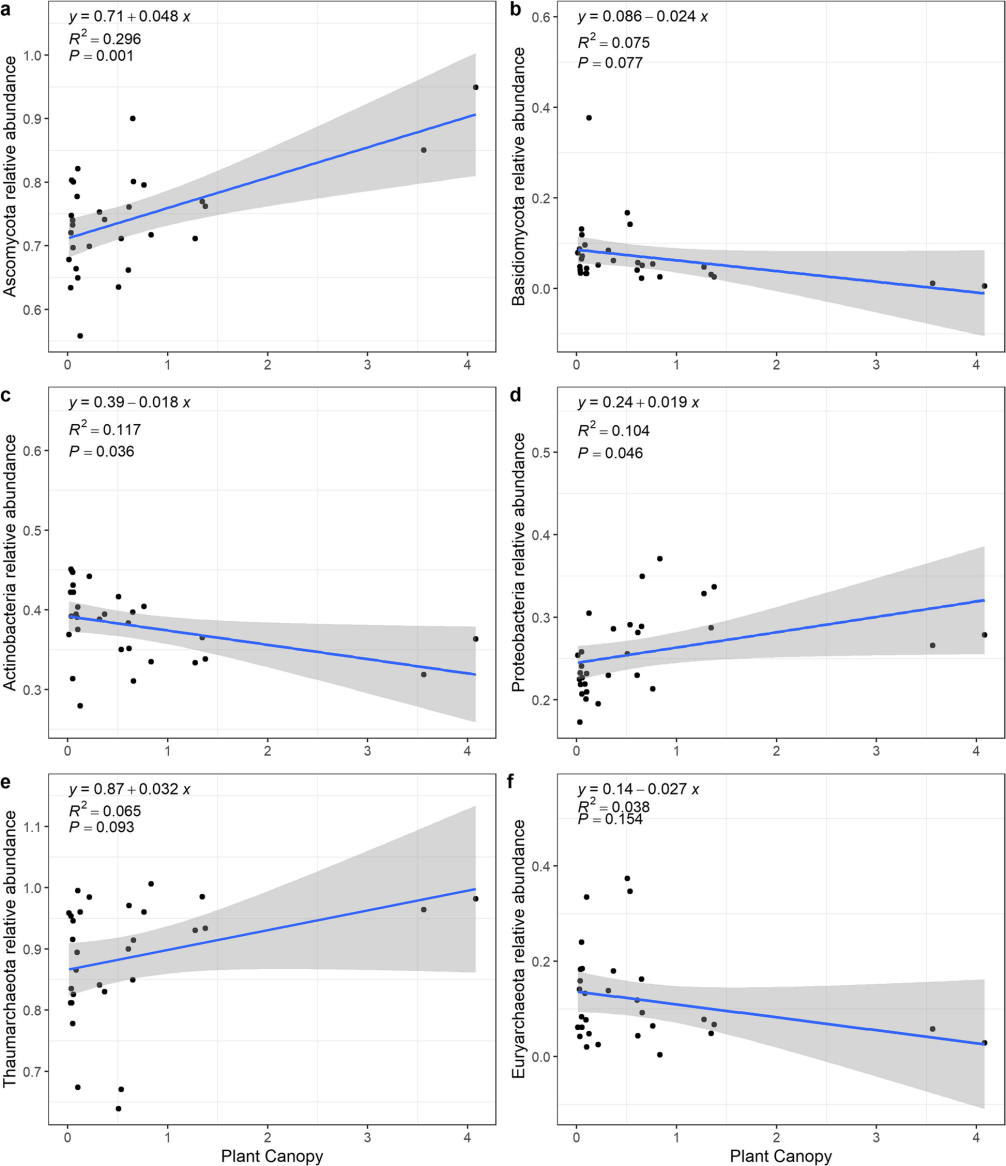
Linear regression analysis of *T. mongolica* canopy size and the soil microbial Shannon index and richness. Panels **a** and **b** are the Shannon-index and richness of the fungal community, panels **c** and **d** are the Shannon-index and richness of the bacterial community, and panels **e** and **f** are the Shannon-index and richness of the archaeal community

The relative abundances of the major phyla in the fungal community beneath the *T. mongolica* shrub canopy from the most to least abundant were Ascomycota, Basidiomycota, Mortierellomycota, Chytridiomycota, Glomeromycota, Cercozoa, Rozellomycota, Calcarisporiellomycota, Olpidiomycota, and Neocallimastigomycota (Figure S1). With increasing shrub canopy size, the relative abundance of Ascomycota significantly increased (*p* = 0.001), while the relative abundance of Basidiomycota showed a decreasing trend (marginally significant, *p* < 0.1) (Fig. 5a, b).

The relative abundances of the major phyla in the bacterial community from high to low were Actinobacteria, Proteobacteria, Chloroflexi, Gemmatimonadetes, Acidobacteria, Bacteroidetes, Cyanobacteria, Firmicutes, Planctomycetes, and Verrucomicrobia (Figure S2). With increasing shrub canopy size, the relative abundance of Actinobacteria significantly decreased (*p* < 0.05), while that of Proteobacteria significantly increased (*p* < 0.05) (Fig. 5c, d).

The phylum composition of the archaeal community was relatively simple, with only four phyla present, and the relative abundances of the phyla from high to low were Thaumarchaeota, Euryarchaeota, Woesearchaeota_DHVEG-6, and Aenigmarchaeota (Figure S3). With increasing shrub canopy size, the relative abundance of Thaumarchaeota marginally increased (*p* < 0.1), while that of the other three phyla did not change significantly (Fig. 5e, f).

### The fertile island effect of T. mongolica shrubs on the soil microbial functional composition

**Fig. 6:**
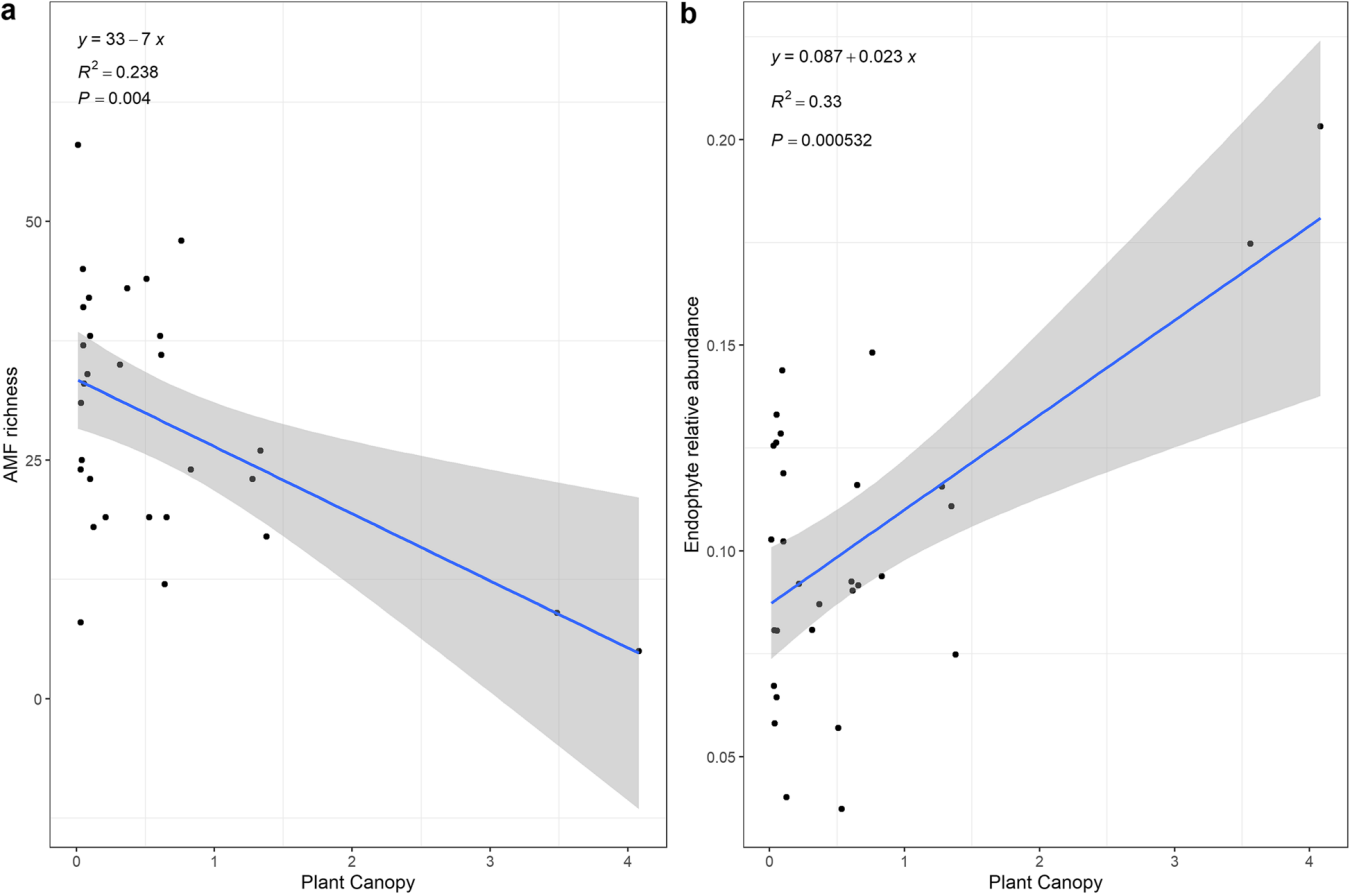
Linear regression analysis of *T. mongolica* canopy size and richness of arbuscular mycorrhizal fungi (**a**) and relative abundance of endophytic fungi (**b**)

The functional groups of the fungi were divided according to their nutrient acquisition modes using FUNguild. The relative abundances of the fungal function groups, from highest to lowest, were as follows: saprotrophs, pathogens, endophytes, parasites, ectomycorrhizal fungi, epiphytes, arbuscular mycorrhizal fungi (AMF), and endomycorrhizal fungi (Figure S4). Except for AMF, there was no significant relationship between the richness of the main functional groups and the shrub canopy size (Figure S5). The richness of AMF decreased significantly (*p* < 0.01) (Fig. 6a); however, the relative abundance of AMF did not change significantly with increasing shrub canopy size (Figure S6). The relative abundance of endophytic fungi significantly increased (*p* < 0.001) with increasing canopy size (Fig. 6b).

**Fig. 7:**
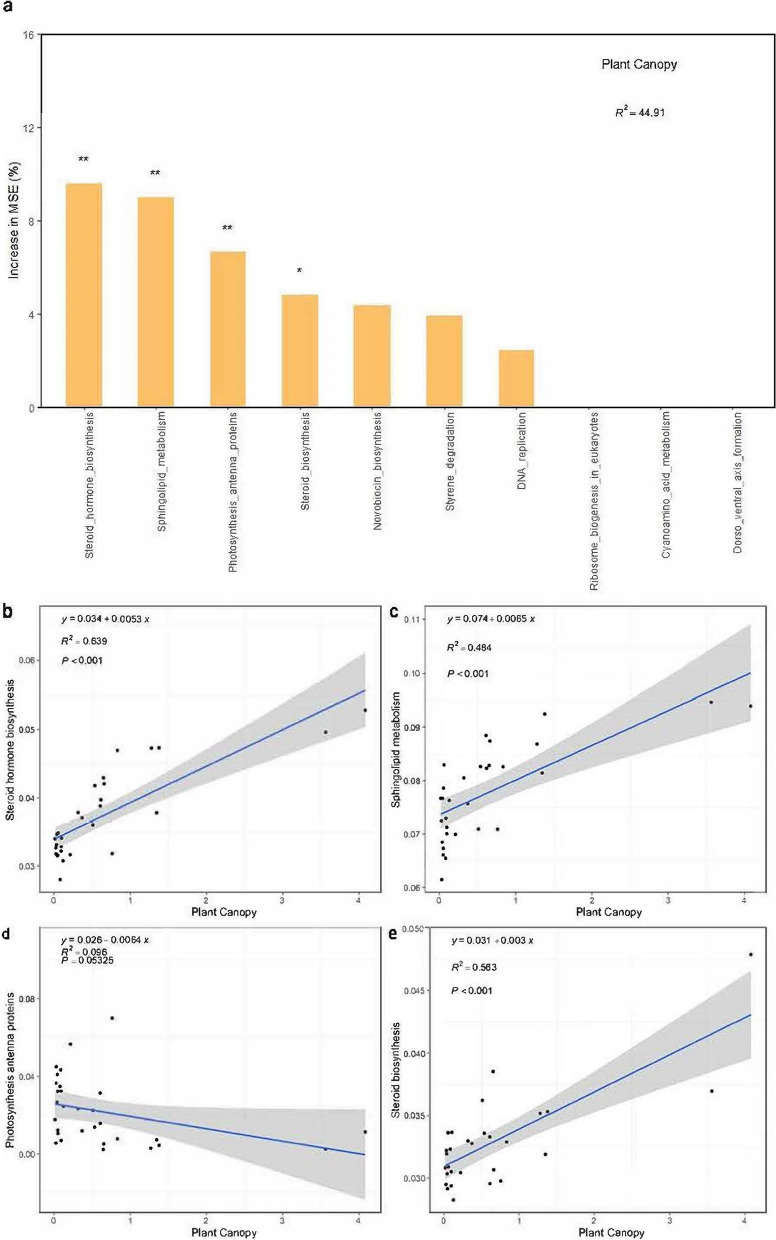
Importance indices of the bacterial functional groups according to *T. mongolica* canopy size (**a**) and correlations between *T. mongolica* canopy size and the main bacterial functional groups (**b**-**e**). An increase in the MSE (%) is the evaluation criterion of feature importance, and the higher the value is, the more important the predictor variable is. * represents significance at *p* < 0.05; ** represents significance at *p* < 0.01

Bacterial functional groups were classified by using KEGG metabolic pathway analysis. The five most abundant bacterial functional groups in descending order of relative abundance were carbohydrate metabolism, amino acid metabolism, metabolism of cofactors and vitamins, metabolism of terpenoids and polyketides, and metabolism of other amino acids (Figure S7). Shrub canopy size significantly affected bacterial functional groups related to the biosynthesis of steroid hormones, sphingolipid metabolism, photosynthesis antenna proteins, and steroid biosynthesis (Fig. 7a). With the exception of photosynthesis antenna proteins, three of the four functional groups are involved in the primary metabolic pathway. The abundance of these three groups significantly increased (*p* < 0.001) with canopy size (Fig. 7b, c and e), while that of the photosynthesis antenna protein group marginally decreased (*p* < 0.1) (Fig. 7d).

**Fig. 8:**
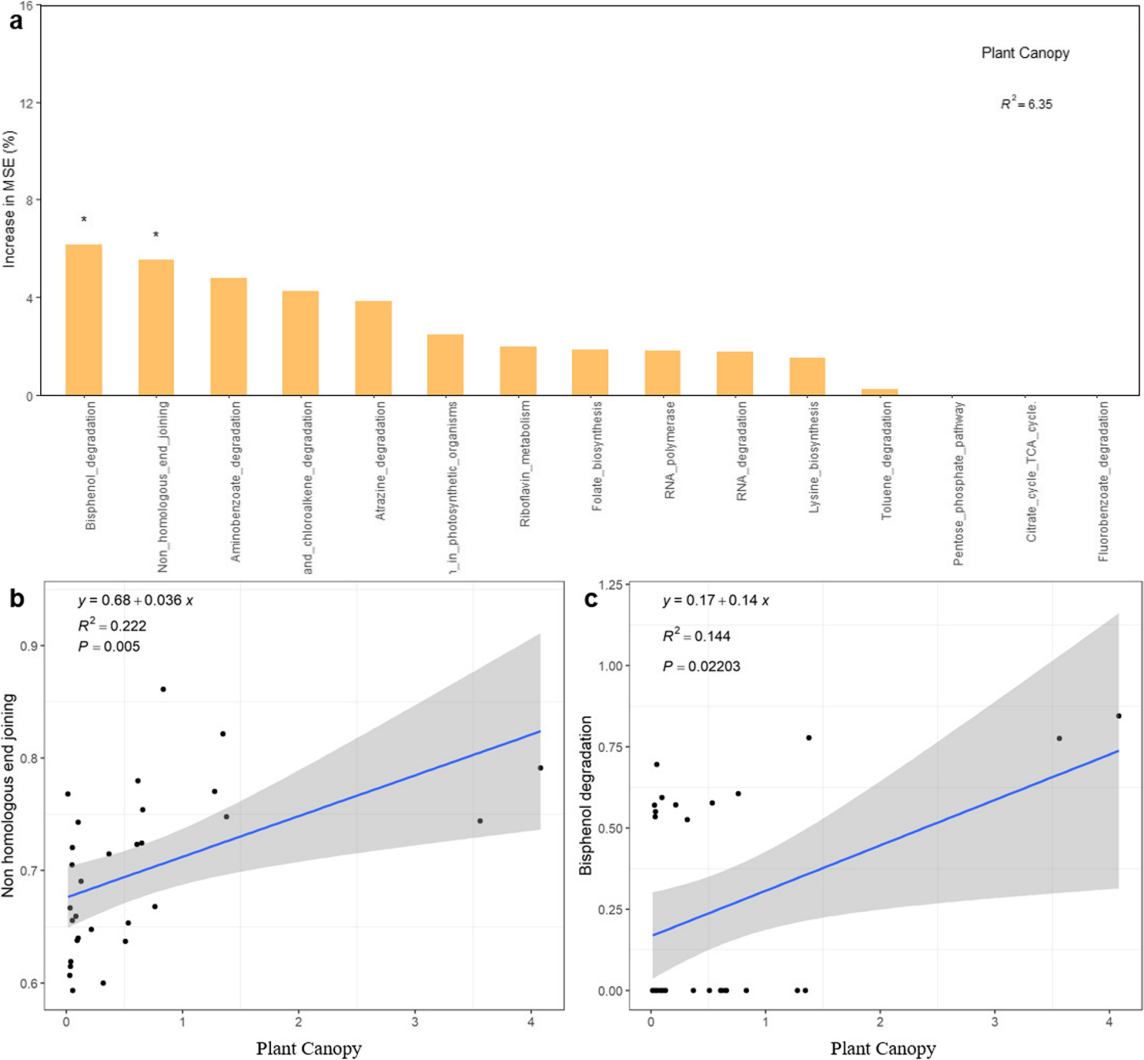
Importance indices of archaeal functional groups in response to *T. mongolica* canopy size (**a**) and correlations between *T. mongolica* canopy size and the nonhomologous end-joining functional group (**b**) and bisphenol degradation functional group (**c**). An increase in the MSE (%) is the evaluation criterion for feature importance, and the higher the MSE is, the more important the predictor variable is. * represents significance at *p* < 0.05; ** represents significance at *p* < 0.01

The archaeal functional groups were classified based on the KEGG metabolic pathways. The five most abundant functional groups were involved in amino acid metabolism, cofactor and vitamin metabolism, carbohydrate metabolism, terpenoid and polyketide metabolism, and exogenous biodegradation and metabolism, arranged in descending order (Figure S8). Shrub canopy size had a significant impact on the nonhomologous end-joining and bisphenol degradation functions (Fig. 8a). These genes are associated with biological genetics and nutrient cycling and are involved in the replication and repair of genetic information processing in the primary metabolic pathway and xenobiotic biodegradation and metabolism functions, respectively. The relative abundance of these two functions increased (*p* < 0.05) with increasing *T. mongolica* canopy size (Fig. 8b and c).

## Discussion

### Fertile island effects of T. mongolica shrubs with different canopy sizes

Plant attributes, such as canopy size, plant height and biomass accumulation, may influence the strength of the fertile island effect through various mechanisms; in particular, greater plant cover improves the efficiency of nutrient redistribution through more developed roots that redistribute nutrients from the interspaces to the vegetated areas and through the accumulation of more litter that releases nutrients to the soil [10, 44–46]. Nutrients are recycled more effectively in the plant‒soil system by soil microbes; for example, studies show that microbial-driven enhancement of soil nitrogen cycling typically associated with high plant diversity levels stimulates productivity [46]. Higher levels of nutrients in the soil beneath the shrub canopy were found in sandy grasslands [35, 37], deserts [10, 47] and savannas [48]. A meta-analysis of paired control data related to shrub-encroached grasslands (SEGs) and non-SEGs collected from 142 studies worldwide showed that shrubs increased topsoil organic carbon [49]. Evidence has shown that the 0–60 cm soil AV, AP, Ak and SOC beneath the *T. mongolica* canopy are significantly greater than those outside the canopy, and the difference is more pronounced at the top of the soil [21], which was similar to the results of this study. As expected, our results showed that there was a fertile island effect beneath the *T. mongolica* shrub canopy. The TN and AVP concentrations beneath the *T. mongolica* shrub canopy were significantly greater than those outside the shrub canopy, and the soil pH beneath the shrub canopy was significantly lower than that outside the canopy. These findings suggested that *T. mongolica* shrubs could change the soil environment.

Moreover, the results of the present study also proved that the size of the *T. mongolica* shrub canopy was related to fertile island effects. The soil TN and AVP beneath the shrub canopy increased with increasing shrub canopy size in the present study (Fig. 2), similar to the findings of *Rhodomyrtus tomentosa* shrubs, which showed that the soil TN and TP beneath a large canopy were significantly greater than those beneath a medium canopy [36]. This might be because the larger the shrub canopy is, the better it is at preserving and acquiring litter under strong wind conditions and thus the more organic matter it is able to contain [50]. Shrubs with larger canopies usually have greater biomass, and thus, they may produce more litter [18, 51]. These litter inputs may decompose, and nutrients recirculate more efficiently within the plant‒soil system [13].

In summary, the soil TN and AVP contents beneath the *T. mongolica* canopy were significantly greater than those outside the canopy, and they increased with increasing *T. mongolica* canopy size. These results partially support our first hypothesis, which is that *T. mongolica* shrubs with different canopy sizes have fertile island effects of different strengths.

### The fertile island effects of T. mongolica shrubs on soil microbes

The abundance, diversity, and composition of soil microbial communities in arid regions worldwide mainly regulate nutrient cycling and litter decomposition rates [31, 52]. The effect of fertile island can recruit soil microbes through higher soil nutrients in the surrounding soil matrix, and more active soil microbial communities can also indirectly enhance the fertile island effect, which in turn enhances ecosystem function by more effectively mobilizing and redistributing nitrogen and other nutrients from the surrounding soil matrix [53–56]. In addition, larger plant canopies provide more suitable habitats for microbial communities because they buffer extreme surface temperatures and retain soil moisture [15]. Our study showed that the fertile island effect of *T. mongolica* shrubs significantly affected the community structure of fungi and bacteria, and some functional groups of soil microbes changed significantly with increasing canopy size. These results supported our second hypothesis, which was that the soil microbial community structure and function beneath the *T. mongolica* canopy are affected by the fertile island effect exerted by *T. mongolica* shrubs, and the strength of this effect varies depending on the canopy size of the shrub. To further investigate the response of soil microbial functional diversity to fertile island size, fungi, bacteria, and archaea were classified into functional groups.

For fungi, the relative abundance of endophytic fungi showed a significant upwards trend with increasing fertile island effect size in the present study (Fig. 6). Endophytic fungi can grow in different organs, histiocytes and intercellular spaces of plants and are distributed in leaves, leaf sheaths, seeds, flowers, stems and roots. These fungi can promote the growth and development of plants by synthesizing auxin or auxin precursors and promoting the absorption of nutrient elements [57–60]. Therefore, the relative abundance of endophytic fungi increased with increasing fertile island effect size, suggesting that the fungal community was more conducive to plant growth. The enrichment of soil nutrients provided a larger niche space for soil microbes and may intensify the competitive relationship between soil microbial communities, resulting in a significant decline in the abundance of AMF with the increase in the fertile island effect in the present study. The change in the relative abundance of AMF was not significant with the increase in the effect of fertile island, indicating that the increase in nutrients due to the increase in the fertile island effect would be more conducive to the survival and reproduction of some AMF. Other studies have shown that plant growth does not increase with the abundance of AMF, and plant growth following inoculation with a mixture of six arbuscular mycorrhizal fungi was similar to that following inoculation with a single AMF [61]. However, there was no direct evidence in this study to indicate whether the changes in AMF were beneficial to the growth and development of *T. mongolica*, which might be related to the specific selection of plants or changes in nutrients; this topic needs to be further explored.

For bacteria, the relative abundance of three functional genes, namely, those involved in steroid hormone biosynthesis, sphingolipid metabolism, and steroid biosynthesis, significantly increased with increasing fertile island effect. The biosynthesis of steroid hormones helps regulate plant growth processes, such as cell elongation, ageing, vessel formation, and stress resistance [62–65]. Plant sphingolipid metabolism plays an important role in plant development, including cell growth, differentiation, reproduction, and response to various stresses [66–68]. The biosynthesis of steroids also plays an important role in plant growth and developmental signalling [65, 69]. The functional traits of microbial genomes can be classified into three main life history strategies: high-yield type (Y), resource acquisition type (A), and stress tolerance type (S) [70]. The increase in functional genes related to the biosynthesis of steroid hormones, sphingolipid metabolism, and steroid biosynthesis and the decrease in functional genes related to photosynthesis antenna proteins reflected the life history strategy of the stress tolerance type (S). This indicated that with increasing *T. mongolica* canopy size, the functional gene expression of bacterial functional groups tended to increase in response to stress more than photosynthesis, which might be more conducive to maintaining the growth and development of *T. mongolica* in harsh environments.

For the archaeal functional groups, enhancing the fertile island effect significantly affected the relative abundance of genes involved in nonhomologous end-joining and bisphenol degradation, both of which increased significantly with increasing fertile island effect. The nonhomologous end-joining function is related to cell ageing, repair, and genetics [71], while the bisphenol degradation function is beneficial to seed germination and the growth of plants, and it has been shown that the higher the concentration of bisphenol is, the stronger the inhibitory effect on seed germination [72, 73]. Low concentrations of bisphenol A promote plant root growth [74, 75]. The upregulation of the expression of these two functional genes also reflected the life history strategy of stress tolerance (S) [70]. Most studies have shown functional redundancy in soil microbial communities [76, 77], which is similar to the redundancy species hypothesis used in biodiversity and stability maintenance mechanism research [78]. Functional redundancy plays a crucial role in the stability of microbial communities. Even if the external environment changes and the structure of the microbial community changes, microbial function remains stable, and the original physiological process is maintained [79]. This study showed that the diversity of archaea significantly decreased with increasing fertile island effect, indicating that the fertile island effect caused by the different sizes of the *T. mongolica* shrub canopy significantly changed the community structure of archaea. However, the percentage of predicted functional groups of archaea associated with changes in the fertile island effect according to the random forest model was only 6.35%, which may be due to functional redundancy in the archaeal community.

## Conclusions

Our results proved that there was a fertile island effect exerted by *T. mongolica* shrubs, resulting in enrichment of soil TN and AVP and changes in the soil microbial community. Additionally, the fertile island effect increased with increasing *T. mongolica* canopy size, which significantly increased the relative abundance of endophytic fungi and biased the expression of functional genes of bacteria and archaea. These changes in soil microbes may be conducive to the survival of *T. mongolica* shrubs in harsh environments. This study comprehensively revealed the impacts of the fertile island effect exerted by *T. mongolica* shrubs on soil and soil microbes and provided a theoretical basis for *T. mongolica* shrub protection, biodiversity maintenance and desertification control. More studies are needed to reveal how the enriched nutrients are used by plants and soil microbes and how the relationship between shrubs and soil microbes changes with the strengthening of the fertile island effect to help shrubs survive.

## Methods

### Study area and plant material

The study was conducted in the Western Ordos National Nature Reserve, Inner Mongolia, China (106°53′1.34’ E, 40°4′54.43’ N, 1080 m a.s.l.). This region has continental monsoon climate characteristics, such as a warm temperate zone, large diurnal temperature difference, little rain, and long periods of sunshine. The annual mean temperature is 7.8–8.1 ℃, the annual mean precipitation is 162–272 mm (concentrated in July and August), the annual potential evaporation is 2470–3481 mm, and the annual mean relative humidity is 43% [80]. It is located in the desert and desert steppe transitional zone and has serious desertification problems; many rare and endangered plant species, such as *Tetraena mongolica* Maxim., *Ammopiptanthus mongolicus* (Maxim. ex Kom.) Cheng f., and *Helianthemum soongoricum* Shenk, are distributed there.

**Fig. 9:**
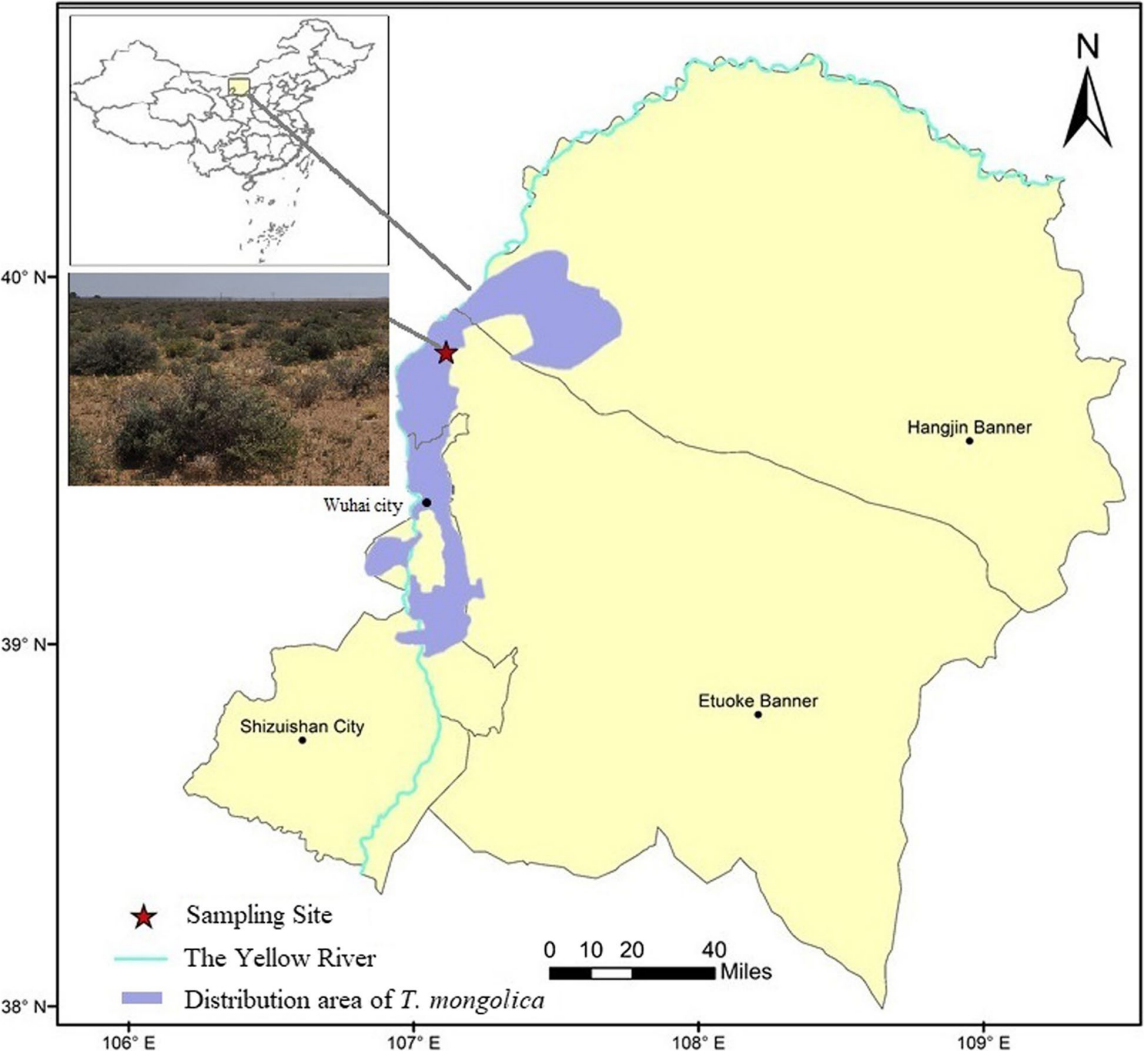
Sampling site and the distribution area of *T. mongolica* in the western Ordos desert

*Tetraena mongolica* Maxim is a relict shrub of the ancient Mediterranean flora and is the only species in the genus Tetraena of the family Zygophyllaceae. It is a small, low, strongly branched shrub, usually 40–60 cm tall. It is distributed only in the western Ordos desert (Fig. 9) and the northern low mountains of Helan Mountain, China, where it plays a critical role as a windbreak and stabilizing the soil.

### Investigation, sampling and laboratory analysis

In September 2020, 30 *T. mongolica* shrubs of different sizes (with canopies ranging from 0.01 m^2^ to 4.08 m^2^; Table S1) were selected for investigation. The longest axis (C1) and the axis perpendicular to C1 (C2) of the plant canopy (m), plant height (cm) and branch number of *T. mongolica* were recorded. The shrub canopy size was calculated as follows:

$$\mathrm{Canopy}=\left(\frac{C_1+C_2}2\right)^2\times\pi\div4$$

Three surface soil (0 ~ 20 cm) cores (5 cm in diameter) were collected around each shrub base and were thoroughly mixed in situ to create one composite sample. Each soil sample was divided into two parts. One part was frozen for microbial sequencing, and the other was used for laboratory analysis.

In the laboratory, soil pH was measured using a pH meter (PB-10, Sartorius, Germany); soil total carbon (TC, g/kg) and nitrogen (TN, g/kg) were determined with an Elemental Analyser (Vario MACRO cube CHNOS Elemental Analyser, Elementar Analysensysteme GmbH, Hanau, Germany); soil total phosphorus (TP, mg/kg), potassium (TK, g/kg) and available potassium (AVK, mg/kg) were determined by inductively coupled plasma-atomic emission spectrometry (iCAP 6300 ICP‒OES Spectrometer, Thermo Fisher, USA); soil inorganic carbon (SIC, g/kg) was determined by a solid-state infrared carbon‒sulfur analyser (multi EA4001, Analytik-Jena AG, Germany); soil organic carbon (SOC, g/kg) was calculated by subtracting SIC from TC; and soil available phosphorus (AVP, mg/kg) was determined by colorimetry using a UV‒visible spectrophotometer (UV‒2550, UV‒visible SPECTRO Photometer, Shimadzu, Japan).

Soil DNA was extracted from the soil samples, and the purification of PCR product DNA was conducted using a QIAquick gel extraction kit (QIAGEN, German) in accordance with the manufacturer’s instructions. This procedure was followed by sequencing on an Illumina MiSeq 300 PE platform (Illumina, San Diego, CA, USA) at Allwegene Technology, Beijing, employing real-time PCR for enhanced accuracy. After rigorous quality control measures, more than 95% of the initial sequence readings were successfully preserved. Sequences containing less than 200 base pairs, displaying a low mass fraction, or comprising ambiguous bases, incomplete primer sequence matches, or barcode label discrepancies (with a sequence ≤ 20) were systematically excluded from the study. For data analysis, QIIME software was utilized. In this study, the operational taxonomic unit (OTU), which serves as an operational definition, was used for the classification of closely related entities in phylogenetic and population genetic analyses. The clustering of sequences into OTUs was performed at a 97% similarity threshold using the UPARSE algorithm. Classification of each OTU was meticulously carried out using the SILVA and UNITE databases [81]. The species diversity of the soil microbes, including bacteria, archaea, and fungi, was calculated based on the number of OTUs in the present study. Reference the cited previous study [43] for more details on the experiment.

### Statistical analysis

All the data analyses in this section were performed in R (version 4.11).

The functional groups of fungal communities were predicted by FUNGuild [82], and Picrust2 was used to predict the functional groups of bacterial and archaeal communities through KEGG pathway enrichment analysis [83]. The Shannon diversity indices of the soil microbes were calculated via the vegan package. The pheatmap package [84] was used to construct a thermal map of the relative abundance of the soil microbes. The ggplot2 [85] and ggpubr [86] packages were used to compare the differences in soil microbial and soil properties beneath and outside the shrub canopy, respectively, with the Wilcoxon rank sum test used to test the significance. Soil property data were log10-transformed. Linear regression analysis was performed between the plant canopy and the soil and soil microbial traits, and a linear regression was drawn with the ggplot2 package.

The random forest package [87] was used to screen the functional groups of bacteria and archaea related to the shrub canopy. First, the functional gene groups with low abundance were filtered to exclude some functional gene groups that may be biased by sequencing or have low classification contributions, and the functional gene groups with relative abundance > 0.1 were selected to construct the random forest model. Second, to facilitate the subsequent evaluation of the performance of the random forest model, the total dataset was divided into a training set (70%) and a test set (30%). The training set was used to train the algorithm to recognize the patterns and relationships between the gene expression and the outcome variable, and the test set was used to evaluate the model's performance. According to the results of tenfold cross-validation, the significant predictor variables of the functional gene groups were evaluated, and 10 and 15 important functional bacterial groups were selected from bacteria and archaea, respectively. The index ‘IncNodePurity’ was used as the importance index of functional gene groups in response to plant canopy size, and the final random forest model was constructed; the greater the value of the "IncNodePurity" indicator was, the greater the importance of this variable. The rfPermute package [88] was used to test the significance of variables, and the ggplot2 package was used to visualize the results.

### Supplementary Information

**Supplementary Material 1.**

## Data Availability

The raw data sequences of soil microorganisms generated during the current study have been submitted to the Sequence Read Archive of the National Center for Biotechnology Information database (https://www.ncbi.nlm.nih.gov/). The BioProject accession numbers are PRJNA1078824, PRJNA1079269 and PRJNA1079702 for soil bacteria, fungi and archaea in 2020, respectively.
