# Supplementary material for: ‘Fertile island’ effects on the soil microbial community beneath the canopy of Tetraena mongolica, an endangered and dominant shrub in the West Ordos Desert, North China
Source: BMC Plant Biol. 2024 Mar 8;24:178. doi: 10.1186/s12870-024-04873-4 (PMC10921620; doi:10.1186/s12870-024-04873-4)
Supplement: Supplementary file 1 — Supplementary Material 1. [file 12870_2024_4873_MOESM1_ESM.docx]

**‘Fertile island’ effects on the soil microbial community beneath the canopy of *Tetraena mongolica*, an endangered and dominant shrub in the West Ordos Desert, North China**

Zhangkai Liu^1, 2, 3^, Yuying Shao^1, 2, 3^, Qingguo Cui ^1, 3^, Xuehua Ye^1, 3, *^, Zhenying Huang^1, 3, *^

^1^State Key Laboratory of Vegetation and Environmental Change, Institute of Botany, Chinese Academy of Science, Beijng, 100093, China

^2^University of Chinese Academy of Science, Beijng, 100049, China

^3^ China National Botanical Garden, Beijing, 100093, China

Zhangkai Liu: liuzhangkai12@163.com; Yuying Shao: shaoyuying22@ibcas.ac.cn; Qingguo Cui: cinkgo@ibcas.ac.cn; Xuehua Ye: yexuehua@ibcas.ac.cn; Zhenying Huang: zhenying@ibcas.ac.cn

***Correspondence author:** Xuehua Ye; Zhenying Huang

**Email:** yexuehua@ibcas.ac.cn; zhenying@ibcas.ac.cn

**Address:** State Key Laboratory of Vegetation and Environmental Change, Institute of Botany, Chinese Academy of Sciences, Beijing 100093, China

**Phone:** 86-10-62836276; **Fax:** 86-10-62836276

Table S1. Traits of *T. mongolica* shrubs and soil properties beneath the canopy.

| No. | Canopy  (m^2^) | Height  (cm) | Branches number | pH | TC (g/kg) | TN (g/kg) | TK (g/kg) | TP (mg/kg) | SOC (g/kg) | SIC  (g/kg) | AVP  (mg/kg) | AVK (mg/kg) |
| --- | --- | --- | --- | --- | --- | --- | --- | --- | --- | --- | --- | --- |
| sp20 | 0.013 | 28 | 5 | 9 | 7.01 | 0.301 | 20.89 | 375.53 | 2.54 | 4.47 | 2.81 | 121.2 |
| sp27 | 0.030 | 15 | 11 | 8.59 | 8.83 | 0.345 | 18.24 | 353.36 | 2.77 | 6.06 | 3.87 | 151.72 |
| sp13 | 0.033 | 14 | 6 | 9.45 | 13.035 | 0.329 | 18.93 | 352.11 | 3.035 | 10 | 4.34 | 125.28 |
| sp8 | 0.035 | 15 | 5 | 9.07 | 19.022 | 0.361 | 18.79 | 360.68 | 3.672 | 15.35 | 5.14 | 153.76 |
| sp9 | 0.038 | 16 | 11 | 9.32 | 13.244 | 0.368 | 18.89 | 376.12 | 3.144 | 10.1 | 5.09 | 183.52 |
| sp11 | 0.049 | 23 | 5 | 9.21 | 7.287 | 0.289 | 18.69 | 332.58 | 2.187 | 5.1 | 2.54 | 110.24 |
| sp7 | 0.051 | 24 | 8 | 9.06 | 17.035 | 0.385 | 18.28 | 421.38 | 4.635 | 12.4 | 9.59 | 190.54 |
| sp18 | 0.053 | 34 | 8 | 9.14 | 6.981 | 0.333 | 18.21 | 330.88 | 1.921 | 5.06 | 3.02 | 117.75 |
| sp5 | 0.055 | 19 | 6 | 8.98 | 22.767 | 0.439 | 19.97 | 349.9 | 5.207 | 17.56 | 10.12 | 215.83 |
| sp19 | 0.083 | 18 | 7 | 9.07 | 8.003 | 0.371 | 18.55 | 373.84 | 2.263 | 5.74 | 2.91 | 113.43 |
| sp3 | 0.093 | 18 | 10 | 9.09 | 18.598 | 0.394 | 18.85 | 358.33 | 6.078 | 12.52 | 11.18 | 232.05 |
| sp10 | 0.102 | 26 | 7 | 9.26 | 7.515 | 0.274 | 18.38 | 340.86 | 1.975 | 5.54 | 4.5 | 158.36 |
| sp1 | 0.102 | 25 | 14 | 8.83 | 9.767 | 0.262 | 18.37 | 338.03 | 2.027 | 7.74 | 4.13 | 219.02 |
| sp6 | 0.126 | 22 | 13 | 9.05 | 20.838 | 0.57 | 19.38 | 365.17 | 6.558 | 14.28 | 7.58 | 238.05 |
| sp14 | 0.216 | 31 | 10 | 9.59 | 15.564 | 0.393 | 18.92 | 337.13 | 3.324 | 12.24 | 6.78 | 164.23 |
| sp24 | 0.317 | 39 | 29 | 8.8 | 12.935 | 0.381 | 18.4 | 361.53 | 3.365 | 9.57 | 8 | 149.04 |
| sp23 | 0.368 | 46 | 39 | 9.16 | 10.869 | 0.417 | 18.7 | 363.35 | 4.079 | 6.79 | 4.87 | 110.88 |
| sp17 | 0.509 | 43 | 37 | 9.16 | 9.591 | 0.389 | 18.59 | 367.72 | 3.381 | 6.21 | 3.87 | 119.79 |
| sp25 | 0.534 | 44 | 67 | 9.36 | 8.378 | 0.347 | 18.03 | 364.25 | 3.668 | 4.71 | 5.93 | 154.14 |
| sp4 | 0.608 | 47 | 42 | 9.09 | 16.937 | 0.407 | 19.56 | 346.82 | 2.647 | 14.29 | 9.27 | 206.25 |
| sp22 | 0.615 | 45 | 141 | 9.09 | 7.285 | 0.318 | 18.03 | 370.26 | 2.375 | 4.91 | 5.99 | 125.15 |
| sp29 | 0.650 | 63 | 51 | 8.62 | 6.607 | 0.308 | 16.99 | 320.53 | 2.647 | 3.96 | 7.31 | 139.08 |
| sp21 | 0.657 | 39 | 145 | 8.65 | 7.987 | 0.442 | 17.75 | 368.82 | 3.637 | 4.35 | 6.25 | 112.95 |
| sp2 | 0.762 | 58 | 124 | 9.06 | 12.025 | 0.399 | 19.17 | 392.07 | 2.565 | 9.46 | 7.47 | 122.73 |
| sp12 | 0.833 | 48 | 89 | 8.59 | 10.52 | 0.581 | 18.36 | 363.9 | 5.79 | 4.73 | 7.58 | 88.92 |
| sp26 | 1.276 | 59 | 56 | 9.21 | 8.548 | 0.42 | 18.46 | 353.14 | 4.308 | 4.24 | 7.47 | 153.12 |
| sp15 | 1.347 | 60 | 95 | 9.21 | 9.054 | 0.424 | 18.62 | 354.03 | 3.924 | 5.13 | 6.83 | 137.8 |
| sp16 | 1.378 | 67 | 56 | 8.59 | 9.984 | 0.508 | 17.47 | 338.96 | 5.494 | 4.49 | 9.96 | 139.46 |
| sp30 | 3.561 | 75 | 327 | 8.76 | 7.707 | 0.49 | 18.11 | 376.66 | 3.617 | 4.09 | 16.85 | 116.29 |
| sp28 | 4.081 | 66 | 242 | 8.84 | 8.233 | 0.503 | 17.15 | 302.64 | 4.563 | 3.67 | 11.6 | 128.86 |

Table S2. Mean (±SE) value of soil properties beneath and outside *T. mongolica* canopy.

|  | pH | TC  (g/kg) | TN  (g/kg) | TK  (g/kg) | TP  (mg/kg) | SOC  (g/kg) | SIC  (g/kg) | AVP  (mg/kg) | AVK  (mg/kg) |
| --- | --- | --- | --- | --- | --- | --- | --- | --- | --- |
| Outside | 9.22  (±0.12) | 10.62  (±3.83) | 0.3  (±0.05) | 19.35  (±0.72) | 350.34  (±22.57) | 3.58  (±1.58) | 7.65  (±3.12) | 3.94  (±1.20) | 136.79  (±23.1) |
| Inside | 9.03  (±0.26) | 11.41  (±4.60) | 0.39  (±0.08) | 18.56  (±0.78) | 357.02  (±22.40) | 3.66  (±3.40) | 7.83  (±4.04) | 6.76  (±3.17) | 149.98  (±39.9) |


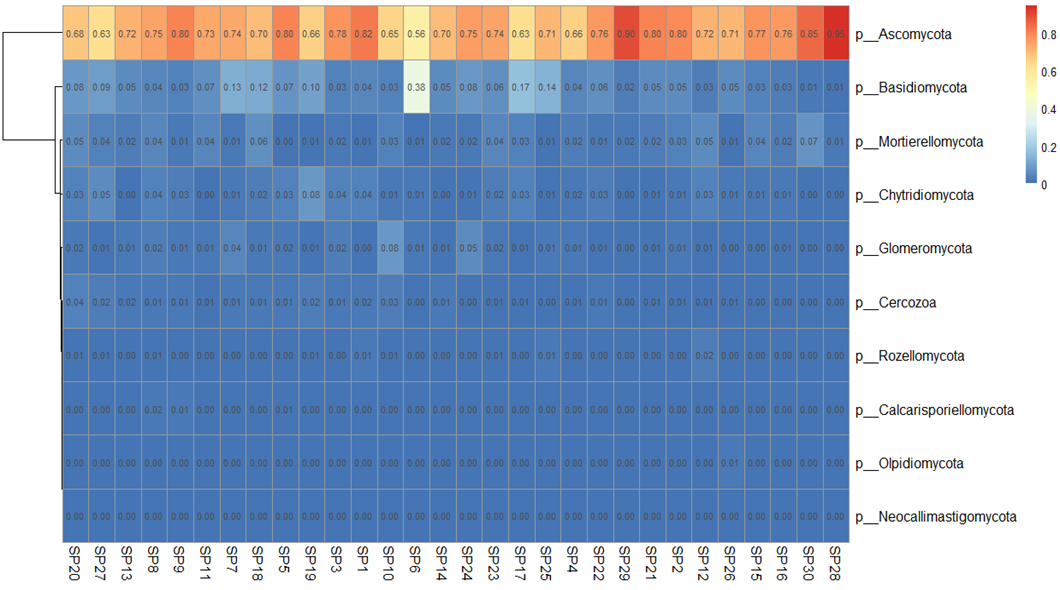


Figure S1. Heat map of relative abundance of main fungal phyla. Each row from left to right, that is sp20-sp28, represents different crown sizes from small to large. Different colors indicate different relative abundances.


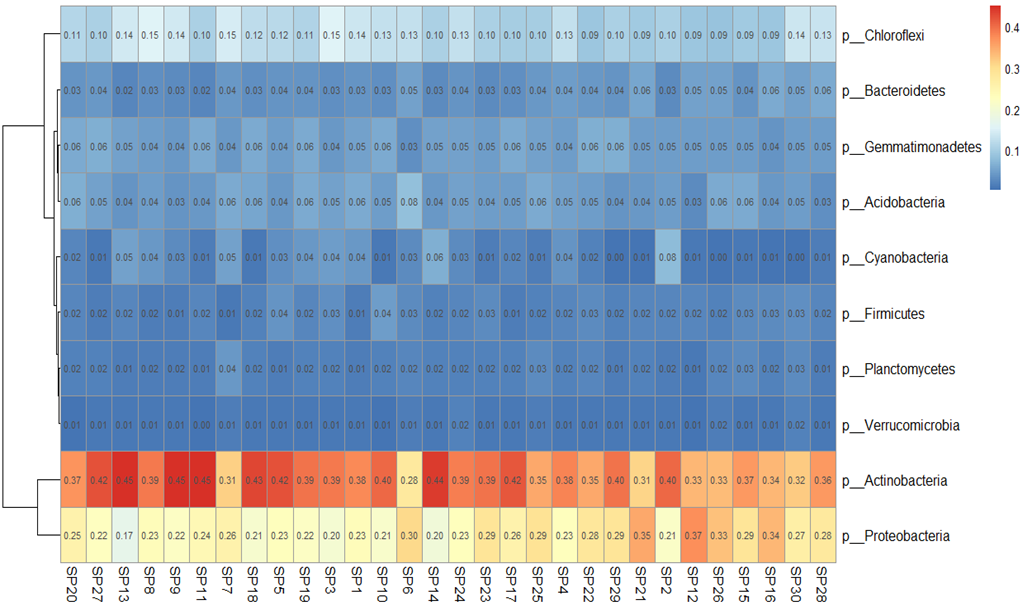


Figure S2. Heat map of relative abundance of main bacterial phyla. Each row from left to right, that is sp20-sp28, represents different crown sizes from small to large. Different colors indicate different relative abundances.


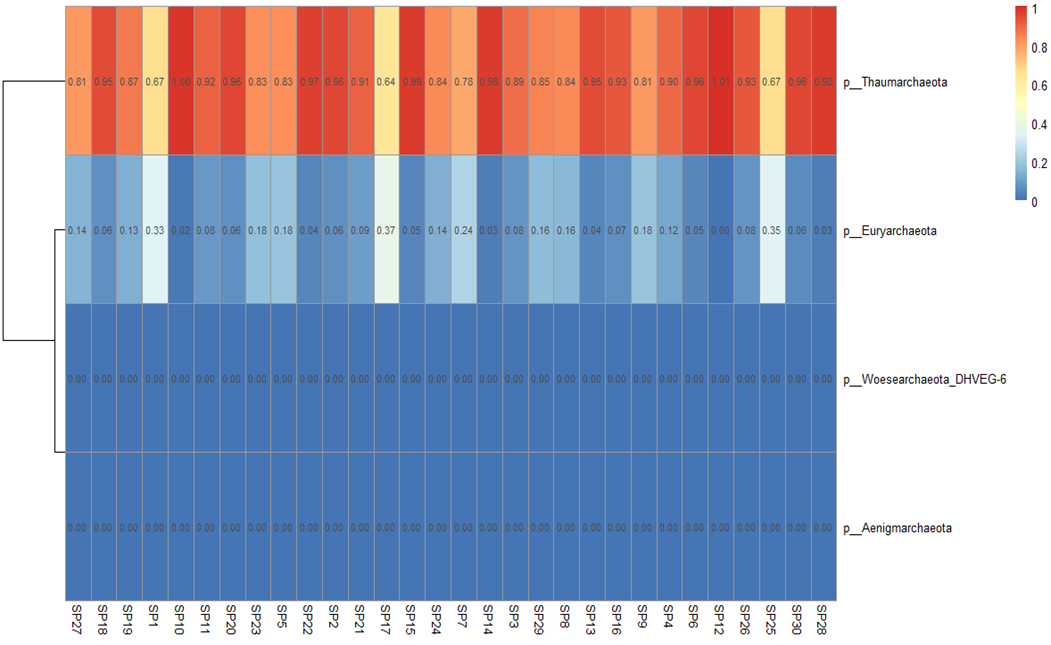


Figure S3. Heat map of relative abundance of main archaea phyla. Each row from left to right, that is sp20-sp28, represents different crown sizes from small to large. Different colors indicate different relative abundances.


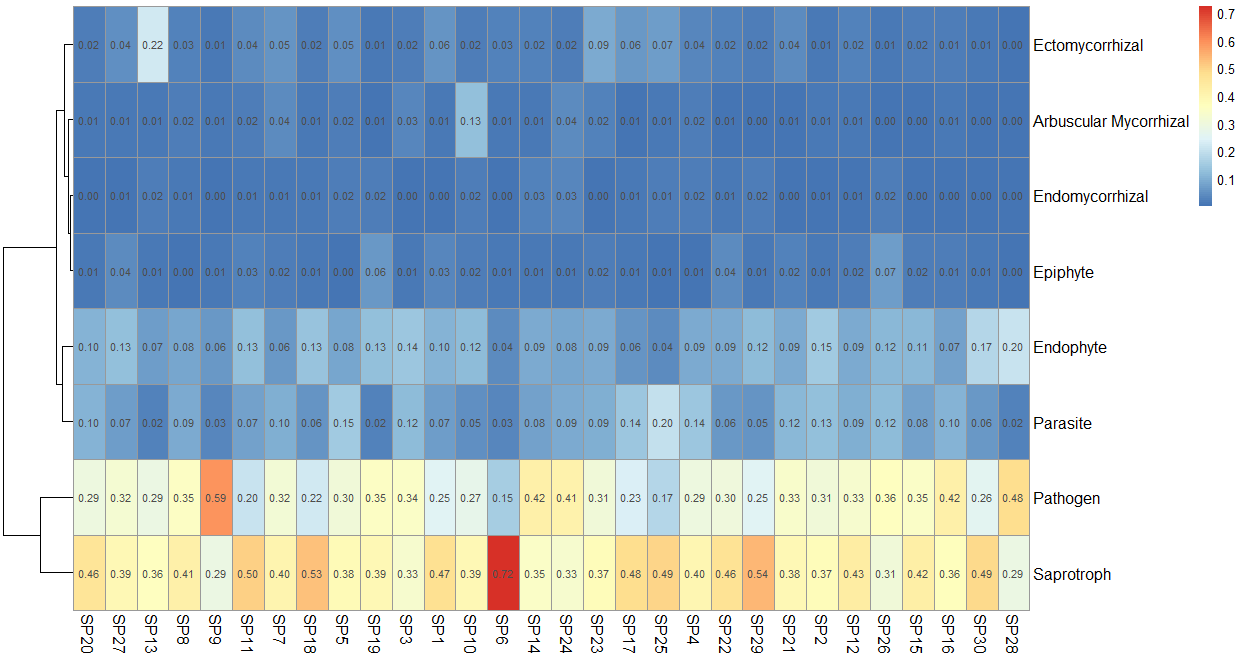


Figure S4. Heat map of relative abundance of main fungal functional groups. Each row from left to right, that is sp20-sp28, represents different crown sizes from small to large. Different colors indicate different relative abundances.

**
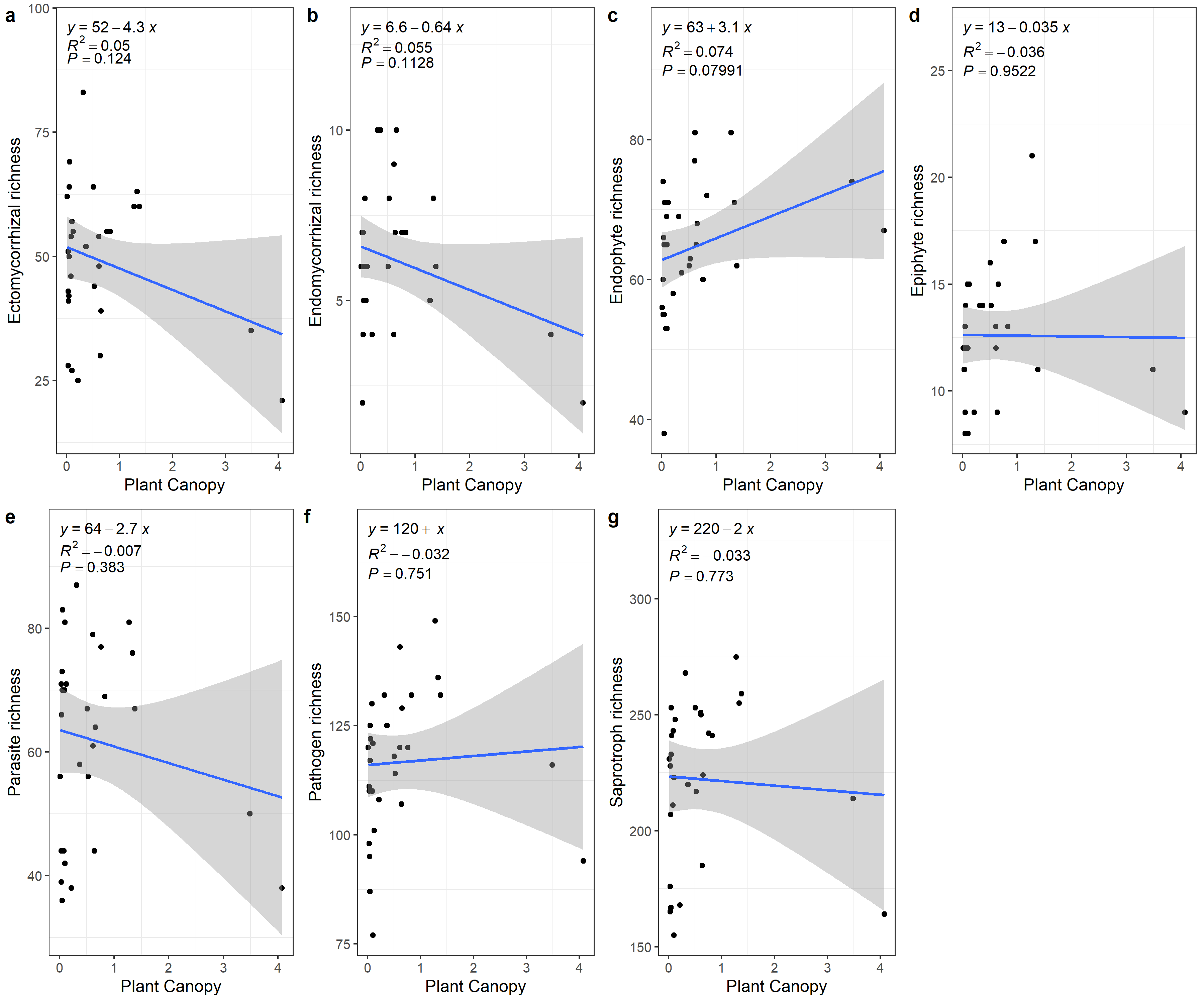
**

Figure S5. Linear regression analysis of *T. mongolica* canopy and richness of main fungal functional groups.

**
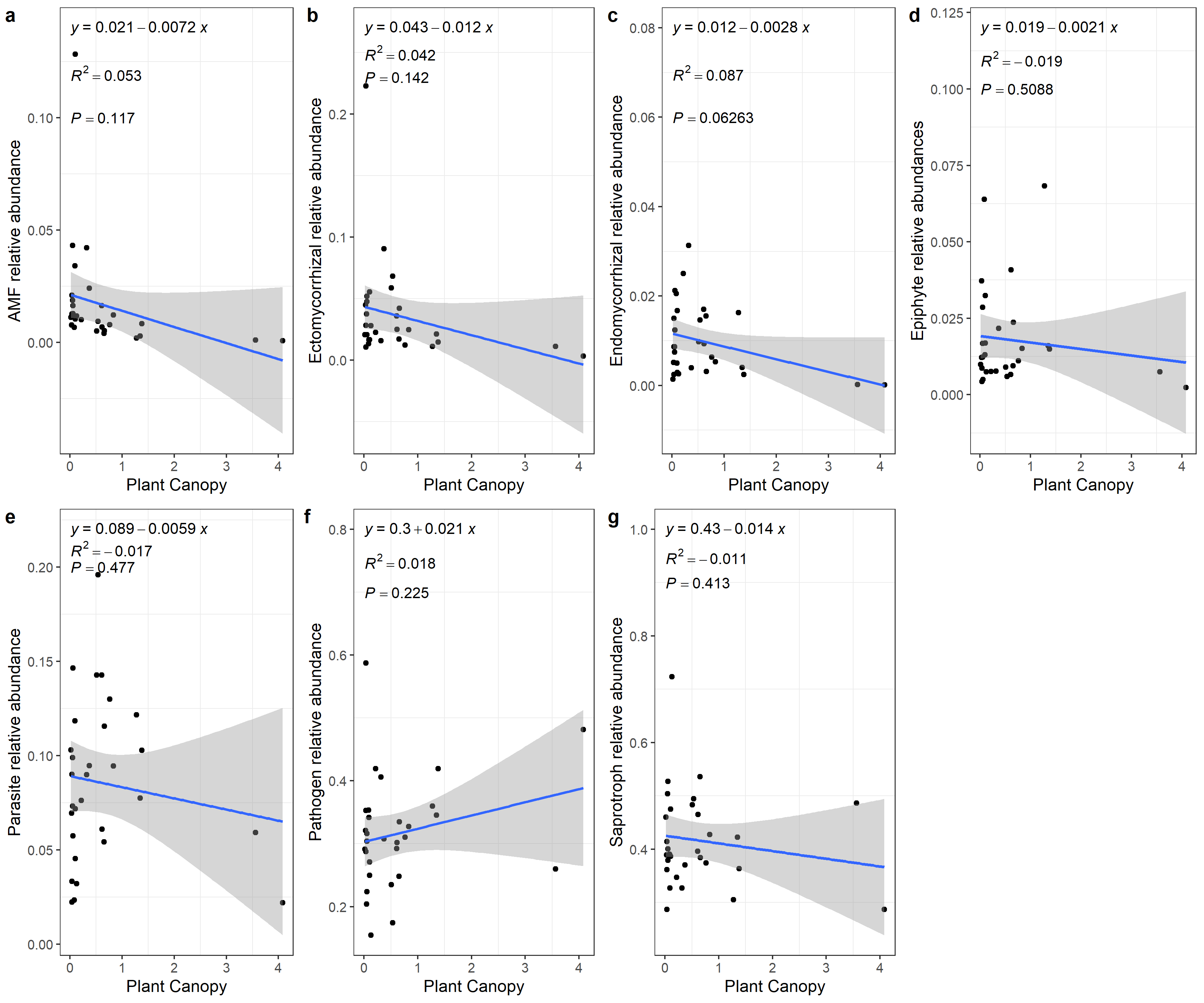
**

Figure S6. Linear regression analysis of *T. mongolica* canopy and relative abundances of main fungal functional groups.


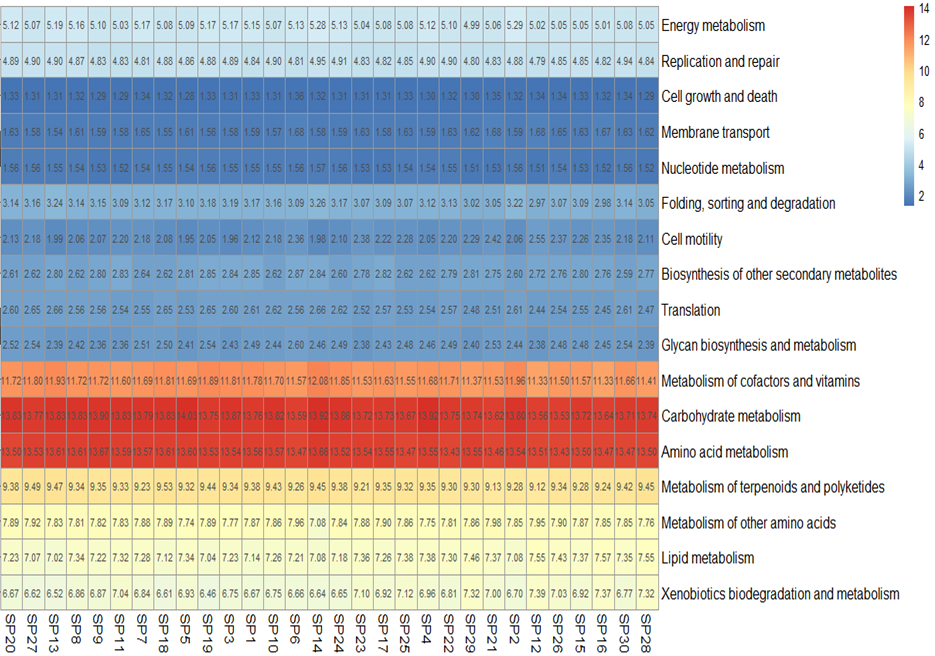


Figure S7. Heat map of relative abundance of main bacterial functional groups. Each row from left to right, that is sp20-sp28, represents different crown sizes from small to large. Different colors indicate different relative abundances.


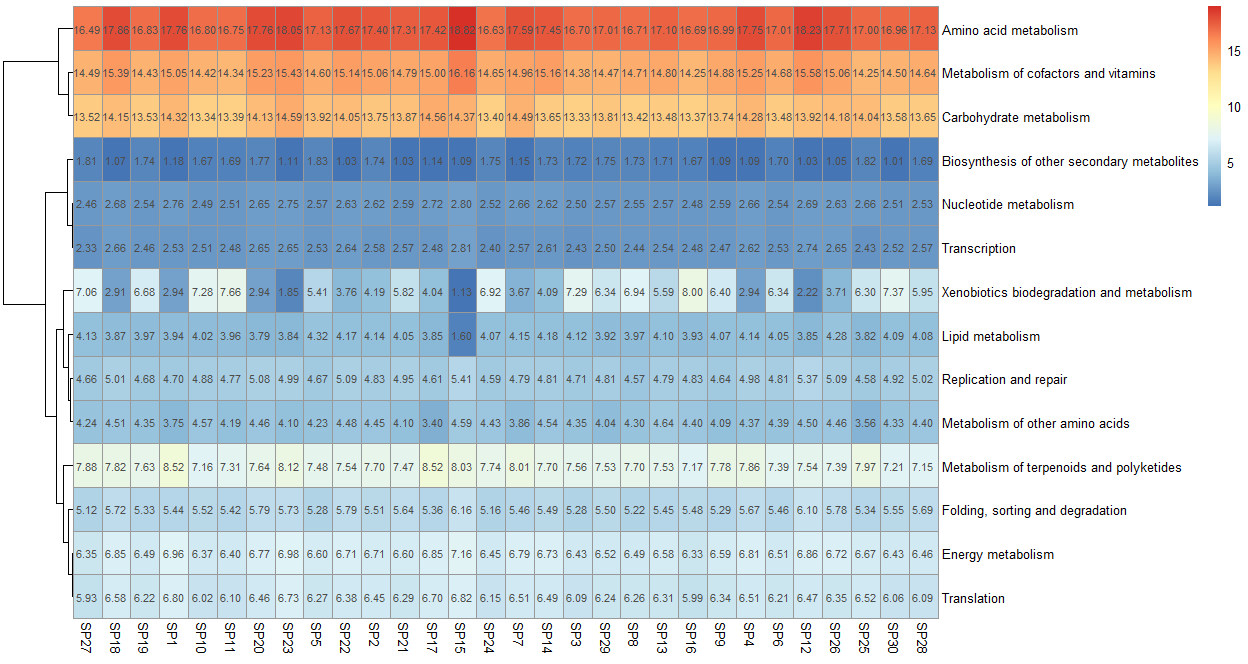


Figure S8. Heat map of relative abundance of main archaea functional groups. Each row from left to right, that is sp20-sp28, represents different crown sizes from small to large. Different colors indicate different relative abundances.
